# Hypoxia-associated prognostic markers and competing endogenous RNA coexpression networks in lung adenocarcinoma

**DOI:** 10.1038/s41598-022-25745-7

**Published:** 2022-12-09

**Authors:** Lecai Xiong, Xueyu He, Le Wang, Peng Dai, Jinping Zhao, Xuefeng Zhou, Hexiao Tang

**Affiliations:** 1 Department of Cardiovascular Surgery, Zhongnan Hospital of Wuhan University, Wuhan, 430071 China; 2 Department of Thoracic Surgery, Zhongnan Hospital of Wuhan University, Wuhan, 430071 China

**Keywords:** Lung cancer, Tumour biomarkers

## Abstract

Lung adenocarcinoma (LUAD) is the most common form of non-small cell lung cancer (NSCLC). Hypoxia has been found in 50–60% of locally advanced solid tumors and is associated with poor prognosis in various tumors, including NSCLC. This study focused on hypoxia-associated molecular hallmarks in LUAD. Fifteen hypoxia-related genes were selected to define the hypoxia status of LUAD by ConsensusClusterPlus based on data from The Cancer Genome Atlas (TCGA). Then, we investigated the immune status under different hypoxia statuses. Subsequently, we constructed prognostic models based on hypoxia-related differentially expressed genes (DEGs), identified hypoxia-related microRNAs, lncRNAs and mRNAs, and built a network based on the competing endogenous RNA (ceRNA) theory. Two clusters (Cluster 1 and Cluster 2) were identified with different hypoxia statuses. Cluster 1 was defined as the hypoxia subgroup, in which all 15 hypoxia-associated genes were upregulated. The infiltration of CD4+ T cells and Tfh cells was lower, while the infiltration of regulatory T (Treg) cells, the expression of PD-1/PD-L1 and TMB scores were higher in Cluster 1, indicating an immunosuppressive status. Based on the DEGs, a risk signature containing 7 genes was established. Furthermore, three differentially expressed microRNAs (hsa-miR-9, hsa-miR-31, hsa-miR-196b) associated with prognosis under different hypoxia clusters and their related mRNAs and lncRNAs were identified, and a ceRNA network was built. This study showed that hypoxia was associated with poor prognosis in LUAD and explored the potential mechanism from the perspective of the gene signature and ceRNA theory.

## Introduction

Lung cancer has high morbidity and mortality, especially advanced or metastatic lung cancer, which often responds poorly to treatment. Non-small cell lung cancers (NSCLCs) account for almost 80% of lung cancers, of which approximately 50% are lung adenocarcinomas (LUADs)^1^. More research to clarify the molecular mechanism and prognostic markers of LUAD is needed. Due to the rise of bioinformatics technology in recent years, numerous studies have emerged to explore the prognostic markers of LUAD from different perspectives. There are relevant tumor marker studies involving the tumor microenvironment^2^, tumor metabolism^3^, cell cycle^4^, DNA methylation^5^, etc., but few studies have been related to hypoxia. This study was conducted to investigate the signature associated with the prognosis of lung adenocarcinoma using hypoxia-related genes as a breakthrough.

Hypoxia is an inherent feature of the tumor microenvironment (TME) that arises from an imbalance between oxygen supply and consumption^6^. An important factor that causes tumor hypoxia is the formation of nonfunctional blood vessels, especially in rapidly growing tumors. Hypoxia status affects multiple cancer cellular responses, such as cell survival, proliferation, epithelial-to-mesenchymal transition (EMT), invasion, immune response, genomic instability, drug resistance, and metastasis^7^. The hypoxia-inducible factor (HIF) family of transcription factors, especially HIF-1α, mediates the expression of multiple genes to drive the adaptation and progression of tumor cells^8^. Many studies have shown that HIF-1α is overexpressed and is associated with poor survival in various solid malignant tumors, such as breast cancer, colon cancer, gastric cancer, and lung cancer^9,10^. Hypoxia status also causes immunosuppression by controlling angiogenesis and favoring immune suppression and tumor resistance, even related to therapy resistance^11^. Therefore, providing markers to assess the degree of hypoxia and hypoxia-related prognosis in lung cancer patients is needed.

In recent years, numerous studies have shown that noncoding RNAs (ncRNAs), including long noncoding RNAs (lncRNAs), microRNAs (miRNAs), and circular RNAs (circRNAs), are involved in human cancers^12^. MiRNA refers to a single-stranded ncRNA with a length of 20 nucleotides that is endogenously expressed and regulates gene expression at the posttranscriptional level. Over the past few decades, numerous experiments have been designed to validate the relevance of miRNAs in disease^13^. A specific model (NCMCMDA, neighborhood constraint matrix completion for miRNA-disease association) has been constructed to predict the correlation between miRNA and disease and has demonstrated good accuracy in diseases such as esophageal and colon neoplasms^14^. LncRNAs are transcripts that are longer than 200 nucleotides and control the expression of genes in the nucleus by interacting with DNA, chromatin-modifying complexes, or various transcriptional regulators^15^. LncRNAs are involved in almost the entire cellular life cycle by different mechanisms and are therefore associated with the development of many diseases, and developing machine learning-based models is one of the most effective ways to explore their roles^16^. The competitive endogenous RNA (ceRNA) hypothesis is one theory linking the function of protein-coding mRNAs to that of ncRNAs, which posits that ceRNAs can impair miRNA activity through sequestration^17^, and lncRNAs are the most reported ceRNAs^18^. The “lncRNA‒miRNA-mRNA” network has been confirmed in many human cancers^19^. There has also been an increasing interest in ceRNA-related research in lung adenocarcinoma in recent years. Most studies have used the TCGA database to find non-coding RNAs significantly associated with LUAD prognosis and have used a multigene regulatory model to identify upstream and downstream related genes and thus construct a network of related ceRNAs network^20–23^. There is also a large body of research exploring the role of ceRNA networks in various pathological processes in LUAD, such as drug treatment sensitivity^24^, tumor immune processes^25,26^, tumor proliferation, migration^27^, novel Cancer Stemness^28^.

In this study, we used 15 hypoxia-related gene expression signatures to characterize the different hypoxia statuses of LUAD samples in The Cancer Genome Atlas (TCGA) and depicted the infiltration of 24 immune cell types and tumor mutational burden (TMB) in LUAD tissues under different hypoxia conditions. Furthermore, the differentially expressed hypoxia-associated miRNAs, lncRNA, mRNAs and related signaling pathways were analyzed. On this basis, a series of prognostic markers related to hypoxia were screened, and a ceRNA network in LUAD was constructed. These results have the potential to further improve the understanding of the regulatory mechanisms under hypoxia in LUAD.

## Methods

### Study cohort

We followed the methods of Zhang et al.^29^. The LUAD gene expression profile and miRNA mature strand expression RNAseq Illumina HiSeq data of TCGA were retrieved from UCSC Xena (TCGA-LUAD)^30^, and the phenotype of LUAD samples was also obtained. The gene expression data included 59 normal samples and 515 tumor samples; among them, 565 samples had complete clinical data. Exchanging the accession number to the ID of miRNA was performed by the miRbase database^31^ and miRBaseVersions.db R package. This study complied with the publication guidelines of TCGA, and ethics approval and informed consent were not needed.

### Classification of hypoxia status

Fifteen hypoxia-related gene expression signatures were selected for our analysis according to published studies: *ACOT7, ADM, ALDOA, CDKN3, ENO1, LDHA, MIF, MRPS17, NDRG1, P4HA1, PGAM1, SLC2A1, TPI1, TUBB6,* and *VEGFA*, which are involved in the hypoxia status^32^. Spearman’s rank correlation was performed to assess the correlation among these genes by the “corrplot” package (sig.level = 0.001), and the PPI network was built using the STRING database (https://string-db.org/). Two different hypoxia status groups (Cluster 1 and Cluster 2) among 515 TCGA-LUAD tumor samples were selected using the ConsensusClusterPlus package (parameters setting: reps = 50, pItem = 0.8, pFeature = 1, clusterAlg = "km", distance = "euclidean"). Principal component analysis (PCA) was performed and visualized by the “limma” package and the “ggplot2” package. The differential expression of these genes between tumor samples and normal samples, Cluster 1 and Cluster 2 were analyzed by the “limma” package with a cutoff P < 0.05 and then visualized by heatmap and vioplot.

### Immune cell infiltration and tumor mutational burden (TMB) analysis

The data of 24 immune cell types and infiltration were acquired from ImmuCellAI Database^33^. The mutation data of LUAD patients were obtained from the TCGA Xena Hub mentioned earlier. The TMB score for each sample was calculated using the following formula^34^: TMB = (total mutation/total covered bases) × 10^6^. The relationship of immune cells/TMB score and hypoxia status was analyzed by the “limma” package with a cutoff of |log2(fold-change)|> 1 and adjusted P value < 0.05^35^.

### Identification of differentially expressed genes (DEGs) related to hypoxia

DEGs between Cluster 1 and Cluster 2 were identified by the “limma” package with a cutoff of |log2(fold-change)|> 1 and adjusted P value < 0.01. Then, the PPI network of DEGs was constructed via the STRING database (https://string-db.org/)^36^, and the crucial subnetwork was selected by the MCODE APP in Cytoscape 3.7.2 according to the following rules: degree cutoff = 10, node score cutoff = 0.2, max depth = 100, and k-score = 2^37^. Gene Ontology (GO) and KEGG pathway enrichment analyses^38^ of subnetworks were performed in the DAVID database (https://david.ncifcrf.gov/tools.jsp)^39^ and visualized by drawing bubble charts.

### Identification of DEGs and construction of prognostic models

We randomly divided 506 samples with complete clinical data into the training cohort and testing cohort. Univariate Cox regression analysis was performed on the training cohort, and then LASSO Cox regression^40^ was employed to select powerful independent prognostic markers with P < 0.05 for OS in LUAD and construct prognostic models. The risk score was calculated by the following formula:

$$ {\text{RS}} = \sum\nolimits_{i = 1}^{n} {Coef\left( i \right)X\left( i \right)} $$

where n represents the gene number in the module, Coef (i) is the coefficient of each gene, and X(i) is the mRNA expression level of each gene. When Coef (i) is less than 0, the corresponding gene has a protective effect on the patient. When Coef (i) is greater than 0, the gene represents the opposite trend for survival. All LUAD samples in the training cohort were divided into high- and low-risk groups. Then, the prognostic values of risk scores in the two groups were analyzed by the Kaplan‒Meier method, and sensitivity and specificity assessments were estimated using receiver operating characteristic (ROC) curves. The relationships between the risk score, immune cell infiltration, TMB score, and clinical characteristics were analyzed in all samples. The prognostic model was verified in a testing cohort and all sample cohorts.

### Differential expression of miRNAs

The differentially expressed miRNAs between Cluster 1 and Cluster 2 were analyzed by the “limma” package with an adjusted P value < 0.05 and |logFC|≥ 1. The prognostic values of differentially expressed miRNAs in LUAD were assessed with Kaplan‒Meier Plotter (https://kmplot.com/analysis/), and the samples were divided into two groups by the best cutoff value by the tool automatically and calculated via Kaplan‒Meier analysis and the log-rank P test for the 120-month OS. We selected the miRNAs related to prognosis and identified the target genes via the mirDIP database (http://ophid.utoronto.ca/mirDIP/index.jsp)^41^. Target genes supported by four or more databases were regarded as candidate genes for further analysis. The intersecting genes of candidate genes and hypoxia-related differentially expressed genes were identified through a Venn diagram. The correlations between intersection genes and corresponding miRNAs were calculated via the StarBase database (https://starbase.sysu.edu.cn/starbase2/index.php)^41^ based on the data from TCGA-LUAD. The prognostic values of selected candidate genes were analyzed by the Kaplan‒Meier Plotter database (http://kmplot.com/analysis/), and miRNA-regulated genes related to the prognosis of hypoxia were identified.

### Identification of target lncRNAs of candidate miRNAs

The target lncRNAs of candidate miRNAs were predicted via the StarBase database^42^. LncRNAs that were negatively correlated with miRNAs (P value < 0.05, correlation coefficient <  − 0.1) and positively correlated with target genes (P value < 0.05, correlation coefficient > 0.1) were selected^41^, and the miRNA‒lncRNA network was constructed via Cytoscape 3.7.2.

### Construction of the ceRNA network and related PPI network

The proteins related to microRNA-regulated genes were predicted in the STRING database, and a network of proteins, miRNAs, and lncRNAs was built through Cytoscape 3.7.2^37^.

### Statistical analysis

**Figure 1:**
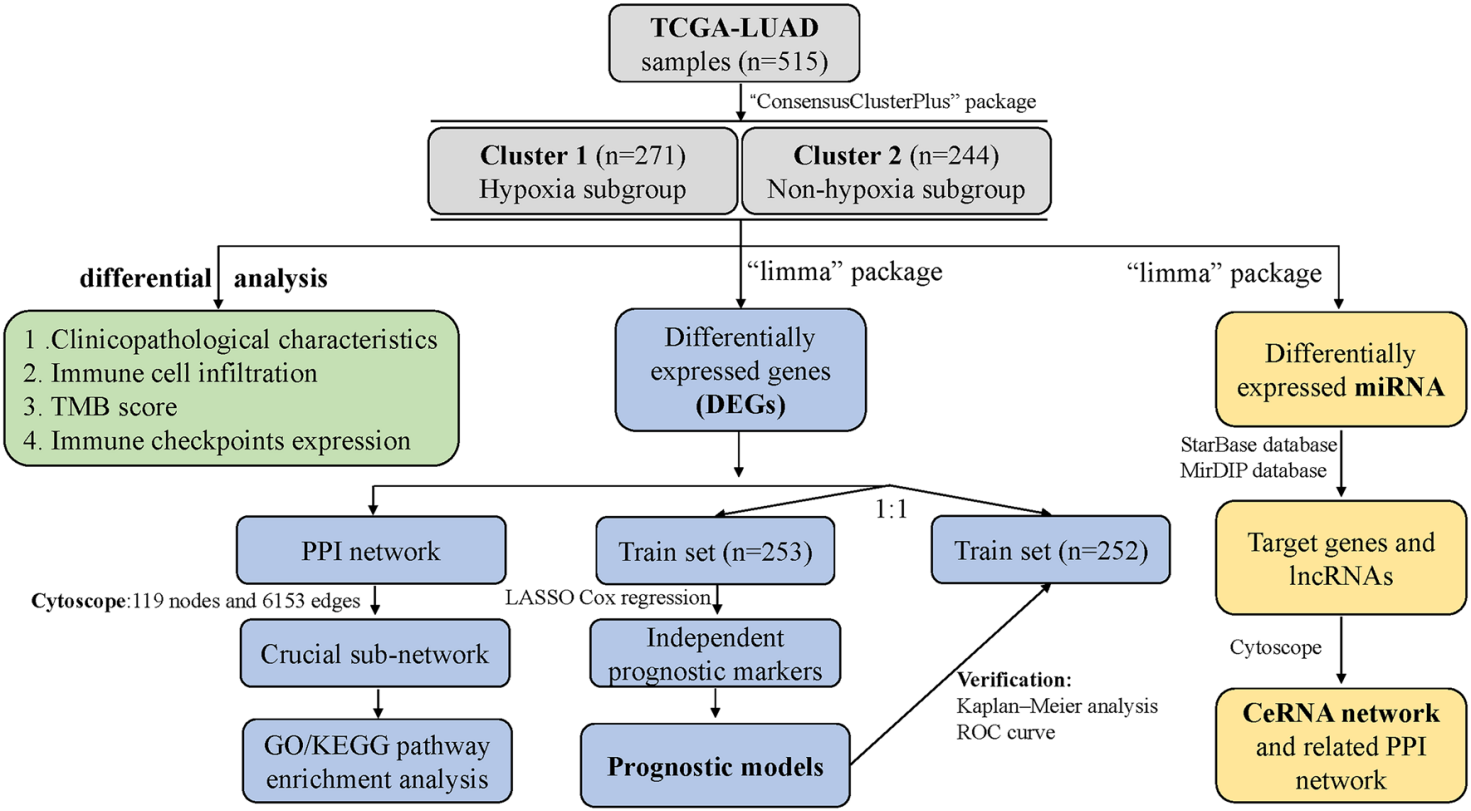
The flowchart of the analyzing process.

All statistical calculations were performed in R software, SPSS or online bioinformatic databases and tools as mentioned. The Wilcoxon test was used to compare mRNA expression, the infiltration score of immune cells and the risk score. The chi-square test was used to compare clinical and pathological parameters and other categorical variables. Differentially expressed miRNAs and mRNAs were calculated by the “limma” R package. The Kaplan‒Meier curve and log-rank P test and univariable Cox and LASSO Cox regression were used to analyze the survival outcomes. ROC curves were utilized to assess the diagnostic effect. The visualization of the data was performed by R 3.6.3, GraphPad 8.0 and Cytoscape 3.7.2. The flowchart of the analysis process is shown in Fig. 1.

## Results

### Consensus clustering identified two clusters of LUAD with different hypoxia statuses

**Figure 2:**
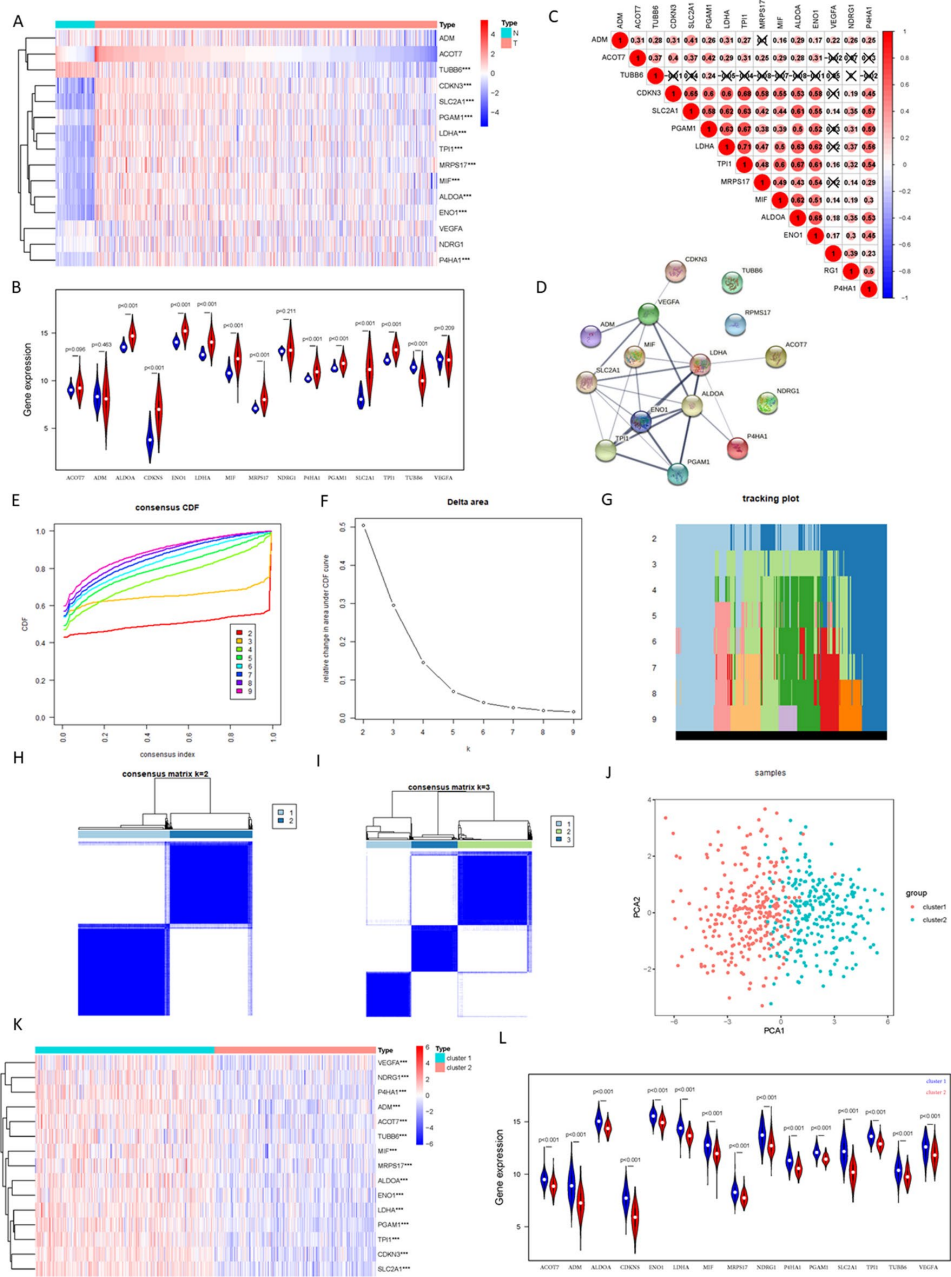
Consensus Clustering identified two clusters of LUAD with different hypoxia status. (**A**, **B**) The heatmap and violin plot of the 15 hypoxia related gene expression signature in TCGA-LUAD tumor and normal samples (Wilcox Test). (**C**) Spearman correlation analysis of the 15 hypoxia related gene expression signature. (**D**) The PPI network of the 15 hypoxia related gene expression signature. (**E**) The CDF value of consensus index. (**F**) Relative change in area under CDF curve for k = 2–9. (**G**) The tracking plot for k = 2 to k = 9. (**H**, **I**) Consensus matrix for k = 2 and k = 3. (**J**) Principal component analysis of the total RNA expression profile. (K-L) The heatmap and violin plot of the 15 hypoxia related gene expression signature in cluster1 and cluster2 (Wilcox Test). *P < 0.05, **P < 0.01, and ***P < 0.001.

Fifteen hypoxia-related gene expression signatures were selected: *ACOT7, ADM, ALDOA, CDKN3, ENO1, LDHA, MIF, MRPS17, NDRG1, P4HA1, PGAM1, SLC2A1, TPI1, TUBB6,* and *VEGFA.* These were highly enriched for hypoxia-regulated pathways^32^. First, we compared the expression differences of these genes in normal tissues and tumor tissues, and the results showed that 10 of them were highly expressed in LUAD (Fig. 2A, B). The interrelationships and correlations were analyzed (Fig. 2C), and the PPI network included 12 genes (Fig. 2D).

According to the expression similarity of these 15 hypoxia-related genes, 515 tumor samples were clustered into different hypoxia statuses by the consensus clustering method. In the CDF curve of the consistent matrix, although the middle section of the CDF curve was flat when K = 2 and K = 3 (Fig. 2E), the interference between subgroups could be minimized when K = 2 (Fig. 2F–I). Thus, two subgroups named Cluster 1 (n = 271) and Cluster 2 (n = 244) were identified. The PCA results suggested that there was a significant distinction between the two clusters (Fig. 2J). To show the hypoxia status of the two clusters more intuitively, we drew a heatmap and a violin map based on the expression of these 15 genes in the two clusters. The results showed that all 15 hypoxia-related genes were upregulated in Cluster 1 (Fig. 2K, L), and we defined Cluster 1 as the “hypoxia subgroup” compared with Cluster 2.

### Clinicopathological characteristics, immune cell infiltration, immune checkpoints, and TMB scores of LUAD patients with different hypoxia statuses

**Figure 3:**
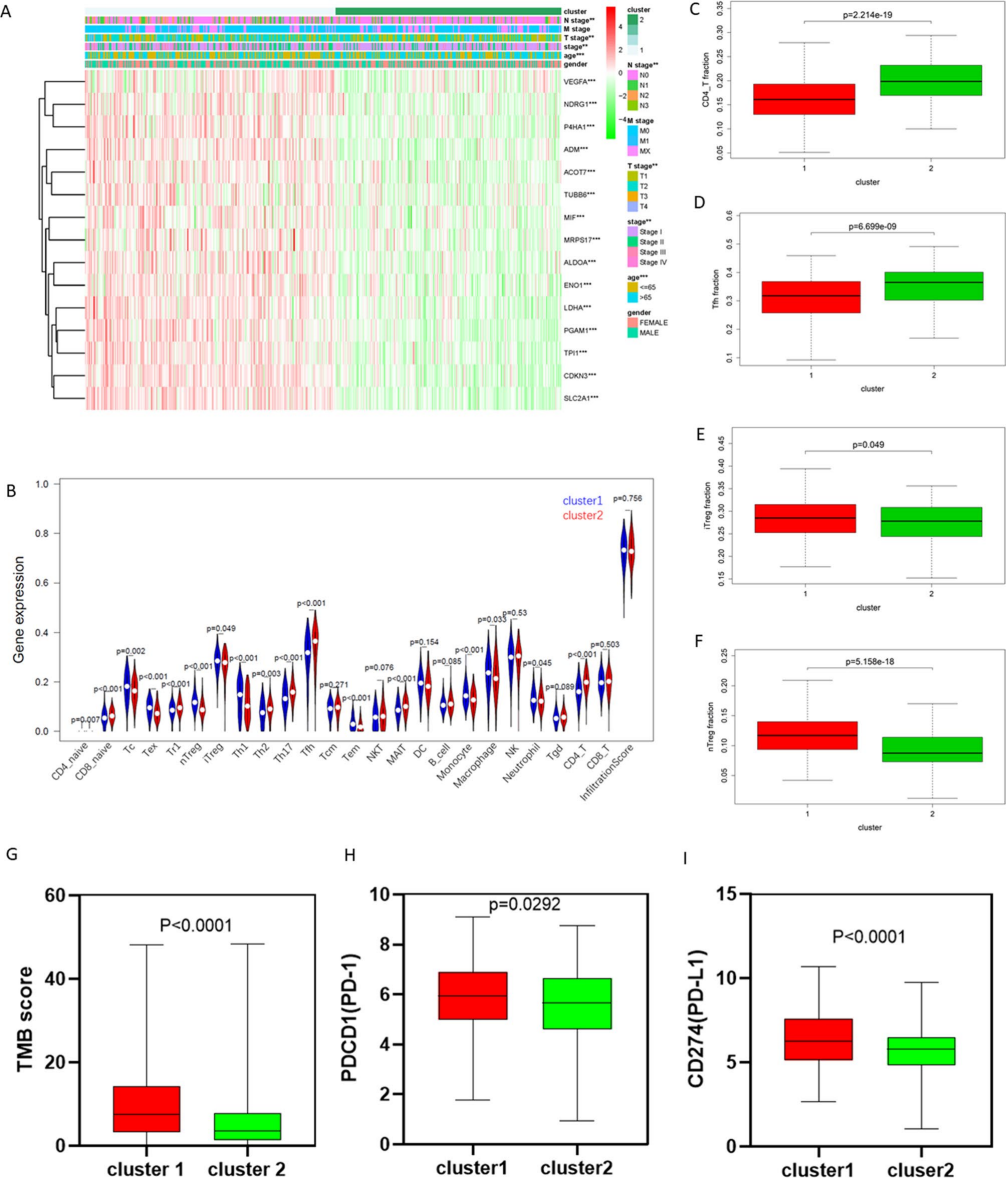
Immune cell infiltration and TMB score of different hypoxia status in LUAD. (**A**) The heatmap of clinicopathological characteristics in cluster 1 and cluster 2. (**B**) The violin plot of infiltration score and 24 immune cell types in cluster1 and cluster2 (Wilcox Test). (**C**–**F**) The infiltration of CD4+ T cell, Thf cell, iTreg cell, and nTreg cell in cluster1 and cluster2 (Wilcox Test). (**G**) The TMB score of cluster 1 and cluster 2. (H-I) The expression of *PDCD1* (PD-1) and *CD274* (PD-L1) in cluster 1 and cluster 2. *P < 0.05, **P < 0.01, and ***P < 0.001.

After excluding samples with incomplete clinical data, the associations between hypoxia status and clinicopathological characteristics were analyzed through the chi-square test in 475 samples. The results showed that the hypoxia status in LUAD was significantly associated with age, tumor TNM stage, T stage and N stage (Fig. 3A, Table 1). The data of 24 immune cell infiltration and infiltration scores in LUAD were obtained from the ImmuCellAI database. The differences in immune cell infiltration and infiltration scores are shown in Fig. 3B, C–F. Compared with Cluster 2, the infiltration of CD4+ T cells and follicular helper T cells (Tfhs) that promote tumor immunity was lower in Cluster 1 (both P < 0.01, Fig. 3C, D), while the infiltration of nTreg cells and iTreg cells that inhibit tumor immunity was higher in Cluster 1 (P = 0.049, 0.000; Fig. 3E, F). The results showed that hypoxia status is not conducive to the tumor immune process. Gene mutations are an important cause of tumorigenesis and development, and TMB is used to predict immune checkpoint blockade therapy efficacy^43^. Hence, we evaluated the difference in TMB scores in Cluster 1 and Cluster 2. The TMB score for each sample was calculated according to the methods, and the results showed that the TMB score was higher in Cluster 1 than in Cluster 2 (Fig. 3G). Furthermore, the expression of PDCD1 (PD-1) and CD274 (PD-L1), the most common immune checkpoints, was higher in Cluster 1 (Fig. 3H, I).

**Table 1:** Clinicopathological characteristics of cluster 1 and cluster 2 in LUAD patients.

Characteristic	Total	Cluster 1(n = 250)		Cluster 2(n = 225)		P Value
		No. of patients	%	No. of patients	%	
**Gender**						
Female	253	124	49.01	129	50.99	0.092
Male	222	126	56.76	96	43.24	
**Age**						
≤ 65	227	139	61.23	88	38.77	< 0.001
> 65	248	111	44.76	137	55.24	
**TNM stage**						
Stage I–II	374	185	49.47	189	50.53	0.002
Stage III–IV	101	65	64.36	36	35.64	
**T stage**						
T1–T2	413	213	51.57	200	48.42	0.004
T3–T4	62	37	59.68	25	40.32	
**M stage**						
M0	317	169	53.31	148	46.69	0.418
M1	22	14	63.63	8	36.36	
MX	136	67	49.26	69	50.74	
**N stage**						
N0–N1	404	205	50.74	199	49.26	0.001
N2–N3	71	45	63.38	26	36.62	

### Identification of hypoxia-related differentially expressed genes (DEGs) and enrichment analysis

**Figure 4:**
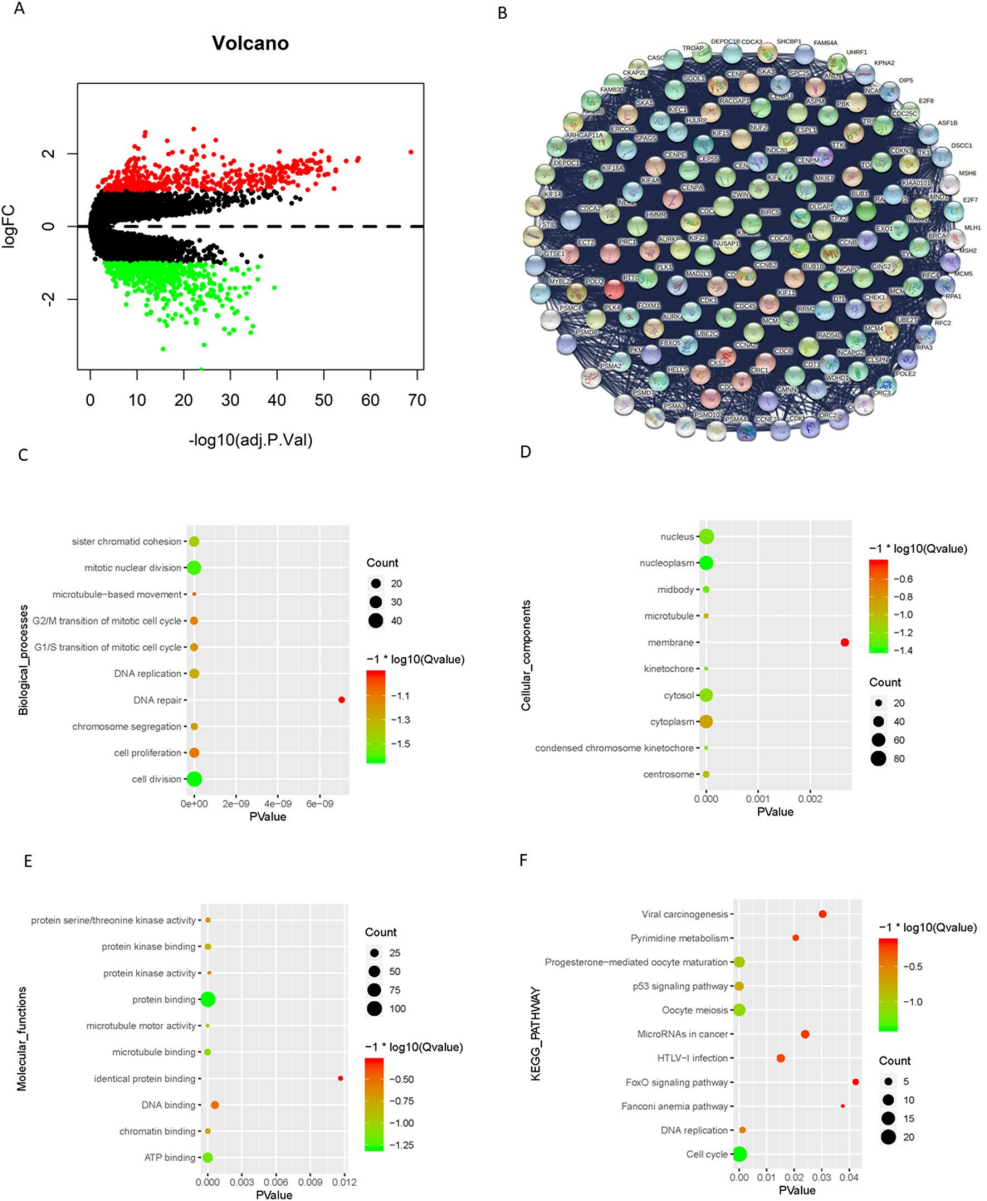
Identification hypoxic-related differentially expressed genes (DEGs) and enrichment analysis. (**A**) Volcano plot for DEGs in cluster1 and cluster2. Red and green dots represent up-regulated and down-regulated DEGs in cluster1 relative to cluster2, respectively (P < 0.01, |logFC|> 1). (**B**) PPI network of a crucial sub-network with 119 nodes and 6153 edges among DEGs. (**C**–**E**) The top 10 items of GO analysis: biological processes, molecular functions and cellular components of the DEGs in the sub-network. (**F**) Pathway enrichment analysis of the DEGs in the sub-network.

Up- and downregulated DEGs (|log2(fold-change)|> 1 and adjusted P value < 0.01) were selected according to cluster. A total of 980 differentially expressed genes were selected, including 440 upregulated and 540 downregulated genes (Fig. 4A). Due to the large number of DEGs, a crucial subnetwork with 119 nodes and 6153 edges was selected by the MCODE app in Cytoscape 3.7.2, and the PPI network was constructed on the STRING database (Fig. 4B). The top 10 items of each GO (biological processes, molecular functions and cellular components) and KEGG pathway analysis are shown as bubble diagrams (Fig. 4C–F).

### Construction of prognostic models based on hypoxia-related DEGs

**Figure 5:**
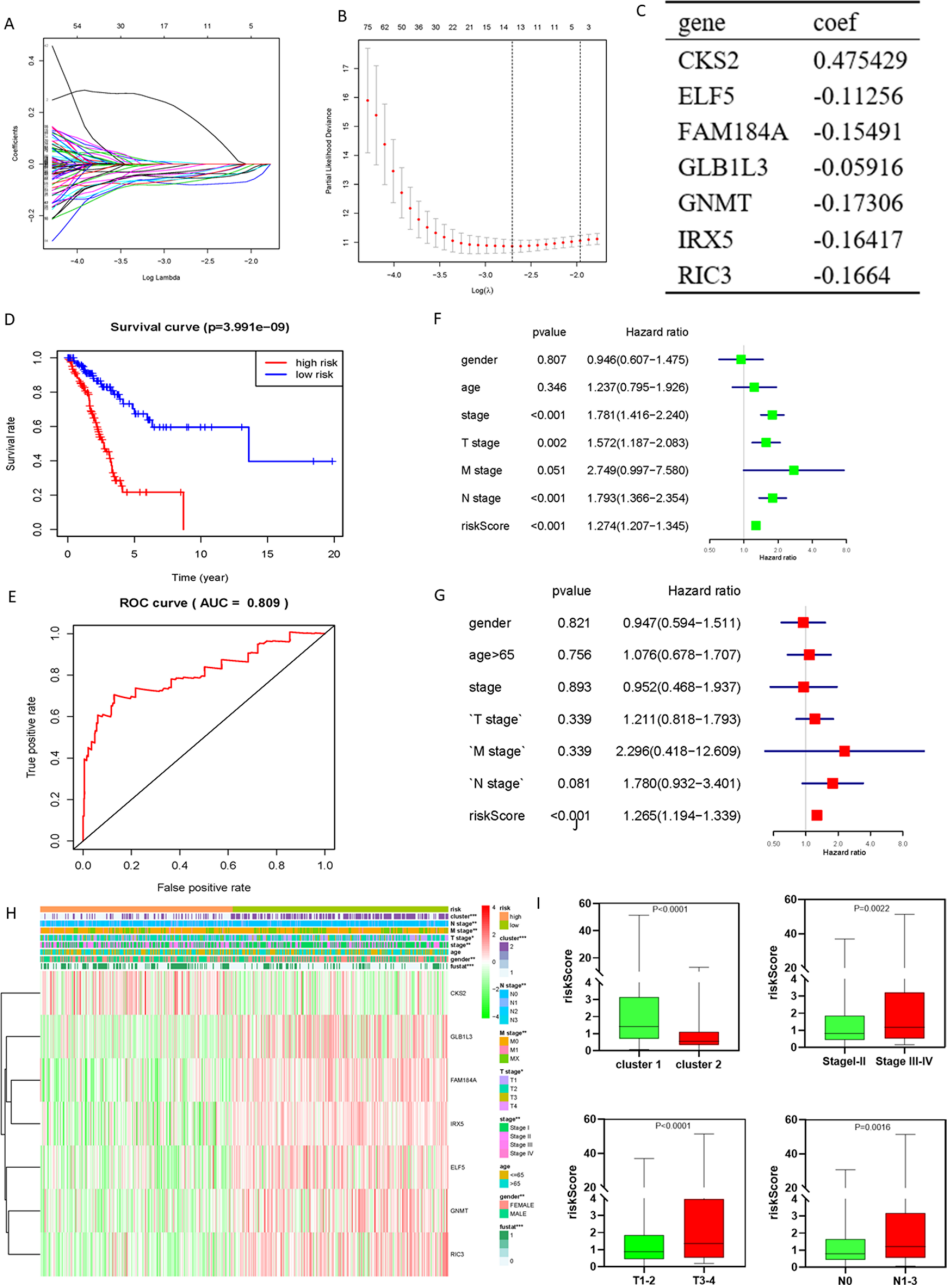
Construction of prognostic models based on hypoxic-related DEGs. (**A**, **B**) LASSO Cox regression was conducted to construct the most powerful prognostic markers. (**C**) Identify 7 powerful prognostic markers and the coefficients by multivariate Cox regression via LASSO in train set (Wilcox Test). (**D**) Kaplan–Meier overall survival (OS) curves for patients in high- and low-risk group in train set (P < 0.001). (**F**) ROC curve and AUC value for risk score in train set. (**F**,**G**) Univariate and Multivariate Cox regression analysis of the associated between clinicopathological features (including risk score) in train set. (**H**) The heatmap shows the expression of the 7 powerful prognostic markers and the distribution of clinicopathological characteristics in high-risk group and low-risk group of all TCGA-LUAD samples (Chi-square Test). (**I**) The relationship between Rick score and cluster status, TNM stage, T stage and N stage (Wilcox Test). *P < 0.05, **P < 0.01, and ***P < 0.001.

Of all 515 tumor samples, 505 contained overall survival (OS) data. The entire set (n = 505) was randomly split into training (n = 253) and testing sets (n = 252) at a 1:1 ratio. The gene expression profiles and the corresponding survival information of LUAD patients in the training set were screened step by step through univariate Cox regression analysis and LASSO Cox regression analysis. A total of 210 genes were identified through univariate Cox regression analysis (Table S1), and 7 genes were ultimately identified as key prognostic hypoxia-related genes (Fig. 5A–C). The training set risk score for OS = (0.475429* expression level of *CKS2*) + (− 0.11256* expression level of *ELF5*) + (-0.15491* expression level of *FAM184A*) + (− 0.05916* expression level of *GLB1L3*) + (− 0.17306* expression level of *GNMT*) + (− 0.16417* expression level of *IRX5*) + (− 0.1664* expression level of *RIC3*).

Samples in the training set were divided into high- or low-risk groups according to the median risk score (1.0). The results of Kaplan‒Meier analysis showed that the high-risk group had a significantly worse prognosis than the low-risk group (Fig. 5D). ROC curves based on the training set were plotted to assess the sensitivity and specificity of the prediction, and the AUC value was 0.809 (Fig. 5E). The results of survival analysis and the AUC of the ROC curve in the testing and entire sets were similar to the above (Fig. S1A, B, E, F). Because the risk score has such a distinctive characteristic, can it become an independent predictor? Univariate and multivariate analyses were performed in the training, testing and entire sets to test whether the risk signature could be an independent predictor. All results showed that the risk score might be an independent risk factor in LUAD patients (all P < 0.05; Figs. 5F, G, S1C, D, G, H).

The expression levels of the 7 genes in all TCGA-LUAD samples (n = 475) were visualized in the heatmap (Fig. 5H), and all TCGA-LUAD samples were classified into high-risk and low-risk groups. The results showed that there were significant differences associated with the risk score regarding cluster (P < 0.0001), TNM stage (P = 0.0022), T stage (P < 0.0001), and N stage (P = 0.0016) (Fig. 5I). The results showed that Cluster 1 (relative hypoxia subgroup) had a higher risk score than Cluster 2, and the higher the tumor stage was, the higher the risk score.

### Identification of prognosis-related differentially expressed miRNAs under hypoxic conditions

**Figure 6:**
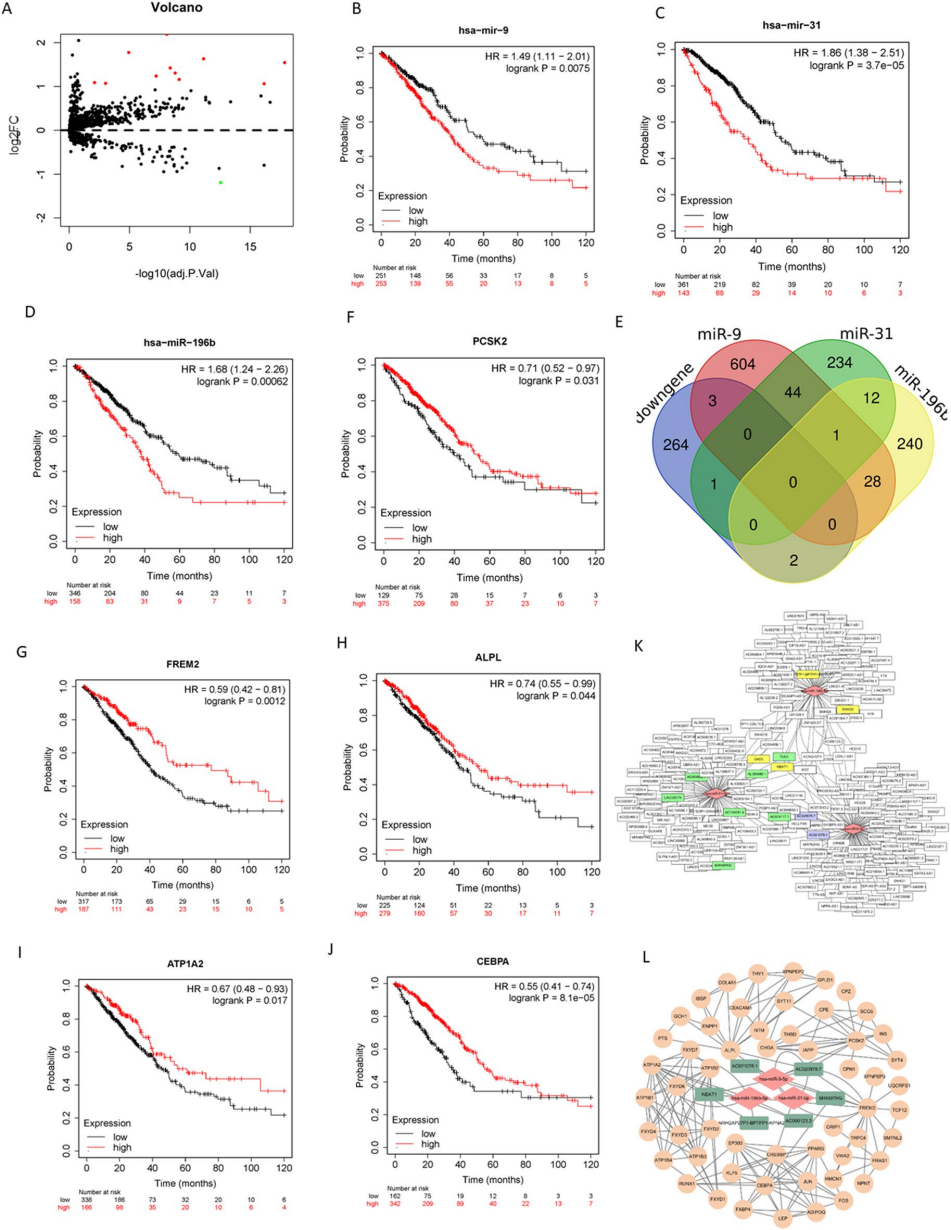
Identification prognosis-related differentially expressed microRNAs and conduction of the ceRNA regulation network under hypoxic status. (**A**) Volcano plot for differentially expressed microRNAs in cluster1 and cluster2. Red and green dots represent up-regulated and down-regulated in cluster1 relative to cluster2, respectively (P < 0.01, |logFC|> 1). (**B**–**D**) The overall survival curves of hsa-miR-196b, hsa-miR-31, hsa-miR-9 in TCGA-LUAD. (**E**) Venn diagrams showing the intersection between predicted target genes of hsa-miR-196b/hsa-miR-31/hsa-miR-9 and DEGs. (**F**–**L**) The overall survival curves of *PCSK2, FREM2, ALPL, ATP1A2, CEBPA* in TCGA-LUAD. (**K**) The microRNA-lncRNA networks of the target lncRNAs of hsa-miR-196b, hsa-miR-31 and hsa-miR-9. (**L**) The ceRNA regulation network based on differentially expressed microRNAs and DEGs under hypoxic status.

Compared with Cluster 2, 11 upregulated miRNAs and 1 downregulated miRNA were identified (Fig. 6A, Table S2). The prognostic value of these 12 miRNAs was evaluated via the Kaplan‒Meier Plotter database. There were 3 upregulated miRNAs associated with OS (hsa-miR-196b, hsa-miR-31, hsa-miR-9). Survival analysis showed that high expression of hsa-miR-196b, hsa-miR-31, and hsa-miR-9 in LUAD was associated with worse OS (Fig. 6B–D; HR = 1.68, 1.86, 1.49, respectively; all P < 0.01).

### Identification of candidate target DEGs regulated by prognosis-related miRNAs

Target genes of miRNAs (hsa-miR-9, hsa-miR-31, hsa-miR-196b) were obtained via the mirDIP database, and those supported by four or more databases were regarded as candidate target genes for further analysis (Fig. S2). The intersection of candidate target genes and the top 50% of downregulated genes was identified through a Venn diagram (Fig. 6E). Three target genes (*PCSK2, FREM2, ALPL*) in hsa-miR-9, two (*ATP1A2, PRSS12*) in hsa-miR-196b, and one (*CEBPA*) in hsa-miR-31 were identified. Except for *PRSS12*, other target genes were related to prognosis, and we defined them as candidate target DEGs (*PCSK2, FREM2, ALPL, ATP1A2, CEBPA*). All candidate target DEGs were downregulated in the relative hypoxia group (Cluster 1), and low expression was associated with poor survival (HR=0.71, 0.59, 0.74, 0.67, 0.55, respectively, all P < 0.05, Fig. 6F–J).

### Hypoxia-related competitive endogenous RNA (ceRNA) regulation network

Target lncRNAs of hsa-miR-196b, hsa-miR-31, and hsa-miR-9 were predicted via the StarBase database, and those that were negatively related to the candidate miRNAs (Fig. 6K) and positively related to the candidate target DEGs were selected (Table 2). Finally, 6 candidate lncRNAs were selected (NEAT1, AC000123.3, ARHGAP27P1-BPTFP1-KPNA2P3, AC020978.7, AC021078.1, MIR497HG). The local protein network between candidate target DEGs (*PCSK2, FREM2, ALPL, ATP1A2, CEBPA*) was constructed, and candidate miRNAs (hsa-miR-196b, hsa-miR-31, hsa-miR-9) and candidate lncRNAs (NEAT1, AC000123.3, ARHGAP27P1-BPTFP1-KPNA2P3, AC020978.7, AC021078.1, MIR497HG) were added (Fig. 6L). In this network, loss of candidate lncRNAs leads to an increase in candidate miRNAs, which suppress the expression of *PCSK2, FREM2, ALPL, ATP1A2,* and *CEBPA*, leading to worse survival in LUAD.

**Table 2:** The correlation coefficient of lncRNA-microRNA and lncRNA-mRNA in TCGA-LUAD.

LncRNA	MicroRNA	Correlation coefficient	P value	mRNA	Correlation coefficient	P value
NEAT1	hsa-miR-196b-5p	− 0.171	P < 0.001	ATP1A2	0.204	P < 0.001
AC000123.3	hsa-miR-196b-5p	− 0.142	P < 0.001	ATP1A2	0.22	P < 0.001
ARHGAP27P1*	hsa-miR-196b-5p	− 0.185	P < 0.001	ATP1A2	0.277	P < 0.001
AC020978.7	hsa-miR-9-5p	− 0.194	P < 0.001	PCSK2	0.137	0.0016
NEAT1	hsa-miR-9-5p	− 0.087	0.0486	FREM2	0.213	P < 0.001
AC021078.1	hsa-miR-9-5p	− 0.097	0.0276	FREM2	0.118	0.0066
AC020978.7	hsa-miR-9-5p	− 0.194	P < 0.001	FREM2	0.321	P < 0.001
AC020978.7	hsa-miR-9-5p	− 0.194	P < 0.001	ALPL	0.182	P < 0.001
MIR497HG	hsa-miR-31-5p	− 0.173	P < 0.001	CEBPA	0.206	P < 0.001

ARHGAP27P1*, ARHGAP27P1-BPTFP1-KPNA2P3.

## Discussion

Tumors are composed of cancer cells and TME, which contains tumor-infiltrating immune cells, cancer-associated fibroblasts (CAFs), endothelial cells, the extracellular matrix, and a wide range of metabolites and cytokines^44^. As the tumor grows, preexisting blood vessels cannot meet the demand, resulting in hypoxia and an acidic environment^45^. Hypoxia can be rapidly sensed by tumor cells, and HIF-1α or HIF-2α activates a gene signature that orchestrates the cellular adaptation to hypoxia^46^. Furthermore, the acidic environment caused by hypoxia impacts the metabolic and functional reprogramming of cancer cells and tumor-associated stromal cells^47^. Therefore, in connection with our research direction, the effect of hypoxia on lung adenocarcinoma is a topic worthy of further discussion. In this study, we not only verified that hypoxia plays an important role in the progression of lung adenocarcinoma from the perspectives of clinical characteristics, immune microenvironment, TMB score, etc., but also constructed a hypoxia-related prognosis model and ceRNA network. This is a relatively complete and multiangle study to explore the impact of hypoxia on the progression of lung adenocarcinoma. According to published studies^32,48^, the 15-gene expression signature (*ACOT7, ADM, ALDOA, CDKN3, ENO1, LDHA, MIF, MRPS17, NDRG1, P4HA1, PGAM1, SLC2A1, TPI1, TUBB6,* and *VEGFA*) that performed best when classifying hypoxia status was selected. These 15 genes make up a common hypoxia signature that will be upregulated and are consistently coexpressed with previously validated hypoxia-regulated genes under hypoxic conditions in various cancers^49^. In this study, based on the differences in the expression of these 15 genes, TCGA-LUAD samples were clustered into different hypoxia statuses according to the expression of the 15 genes. All 15 genes were upregulated in Cluster 1 (Fig. 2K–L), which was defined as the “hypoxic subgroup”. In analyzing the relationship between the hypoxia status of lung adenocarcinoma and clinicopathological characteristics, we found that hypoxia is associated with worse TNM staging, suggesting that hypoxia is associated with poor prognosis in LUAD (Fig. 3A, Table 1).

Numerous studies have indicated that the hypoxia of the TME promotes tumor immunosuppression and resistance to immunotherapy^50^. The hypoxic tumor region can recruit immunosuppressive cells such as myeloid-derived suppressor cells (MDSCs), tumor-associated macrophages (TAMs), tumor-associated neutrophils (TANs) and Tregs and negatively affect the activation of CD8+ T cells and CD4+ T cells^51^. Hypoxic cancer cells, via HIF-1α, secrete the chemokine CCL28, which recruits CXCR10+ Tregs into tumors^52^. TGF-β is a cytokine highly abundant in hypoxic regions of the tumor^53^, leading to the development of TANs. TGF-β induces the production of Foxp3 and RORγt in CD4+ T cells, which induces the differentiation of Tregs and enhances immunosuppression^54^. In this study, the infiltration of 24 immune cell types was compared in Cluster 1 and Cluster 2. The results showed that the infiltration of CD4+ T cells and Tfh cells was lower, while the infiltration of nTreg cells and iTreg cells was higher in Cluster 1 (Fig. 3), indicating that there is an immunosuppressive state in Cluster 1. A study in breast cancer showed that hypoxia increased TMB by driving genome instability and altering DNA damage repair pathways^55^. The same phenomenon was observed in this study. The TMB score and PD-1 and PD-L1 expression in Cluster 1 were significantly higher than those in Cluster 2 (Fig. 3H), indicating an immunosuppressive status.

Under hypoxic conditions, tumor cells activate multiple adaptive pathways to promote the evolution of a more aggressive tumor phenotype, including the activation of DNA damage repair proteins, altered metabolism, and decreased proliferation^56^. In this study, the differentially expressed genes between Cluster 1 and Cluster 2 were identified, and GO/KEGG analysis was performed. The results showed that the differentially expressed genes related to metabolism, such as ATP binding, cell cycle and proliferation, such as cell division, cell proliferation, G1/S/G2/M transition of the mitotic cell cycle, etc., were related to DNA damage repair, such as DNA replication and DNA repair, and immune regulation, such as the FoxO signaling pathway (Fig. 4). HIF is stably expressed under hypoxia and promotes angiogenesis through VEGF-A, glycolysis, and pH control through CA-IX^57^. There is extensive evidence showing the downregulation of numerous proteins involved in homologous recombination, mismatch repair, base excision repair, and nucleotide excision repair under hypoxic conditions^58^. The FoxO signaling pathway is a pivotal regulator of Treg cell function, which promotes immune suppression^59^. Furthermore, numerous DEGs were enriched in microtubule-based movement, protein kinase activity, the p53 signaling pathway, microRNAs in cancer, etc., which are all related to tumor invasion and metastasis. These underlying mechanisms together lead to the negative impact of hypoxia on tumor prognosis. To better predict the prognosis of patients, we constructed a risk signature containing 7 genes by univariate Cox and LASSO Cox regression analysis, which showed a good predictive ability (Fig. 5).

MiRNAs and lncRNAs have been identified as key regulators of gene expression in various biological and pathological processes^60^. With the development of computational biology and sequencing technologies, and the improvement of computer deep learning capabilities^61^, numerous miRNAs and lncRNAs have been rapidly discovered, and gradually, the interaction between lncRNAs and miRNAs has received increasing attention, but little is known. Many studies have built multiple models to achieve the mutual prediction of lncRNAs and miRNAs^42,62^, which is crucial to improving ceRNA network theory. Currently the two main types of prediction models are namely network algorithm and machine learning-based model^63^. We identified potential ncRNA regulatory pathways involving miRNAs, lncRNAs and mRNAs based on ceRNA theory and mature prediction models and built a PPI network that might promote the development of LUAD (Fig. 6). Studies have shown that hsa-miR-196b is a potential biomarker in LUAD^64^, an *ATP1A2* mutation is found in pulmonary carcinoid tumors and is involved in multiple biological processes, such as cellular metabolism and immune regulation, and *NEAT1* functions as a competing endogenous lncRNA in multiple tumors^65^. According to the results of this research, *NEAT1,* as a sponge of hsa-miR-196b, alleviates its repression of *ATP1A2* and regulates multiple biological processes in LUAD. Similarly, hsa-miR-31 and its predicted target lncRNA (*MIR497HG*) and mRNA (*CEBPA*) are all involved in multiple tumors^66^, and there is also a competing endogenous molecule between them in LUAD according to our results. The levels of hsa-miR-9 correlate with tumor grade and metastatic status^67^, and its upregulation leads to enhanced NSCLC cell invasion and adhesion via the regulation of multiple pathways^68^. According to this study, samples in TCGA-LUAD with high expression of hsa-miR-9 have worse survival, and various lncRNAs (AC020978.7, NEAT1, AC021078.1) and mRNAs (*PCSK2, FREM2, ALPL*) are all related to survival.

In conclusion, this study explored the role and potential mechanism of hypoxia in LUAD from the perspective of gene signature and ceRNA theory in silico analyses. The results showed that hypoxia promoted tumor progression and immunosuppressive status through multiple pathways, and the regulatory effect of ceRNA theory on LUAD was also observed. However, it is undeniable that our research still has some limitations. All our results are based on in silico analyses of TCGA and some website analyses. More functional experiments are needed to verify the results of this research, which will be the focus of our future studies.

## Supplementary Information

Supplementary Figures.

Supplementary Table S1.

Supplementary Table S2.

## Data Availability

The datasets generated and analyzed during the current study are available in the TCGA (https://portal.gdc.cancer.gov/ (Selection criteria: homepage, select Repository, Files Types select RNA-Seq, Cases types select TCGA-LUAD in bronchus and lung).
