# Supplementary material for: Hypoxia-associated prognostic markers and competing endogenous RNA coexpression networks in lung adenocarcinoma
Source: Sci Rep. 2022 Dec 9;12:21340. doi: 10.1038/s41598-022-25745-7 (PMC9734750; doi:10.1038/s41598-022-25745-7)
Supplement: Supplementary file 1 — Supplementary Figures. [file 41598_2022_25745_MOESM1_ESM.docx]

***Supplementary Figures***


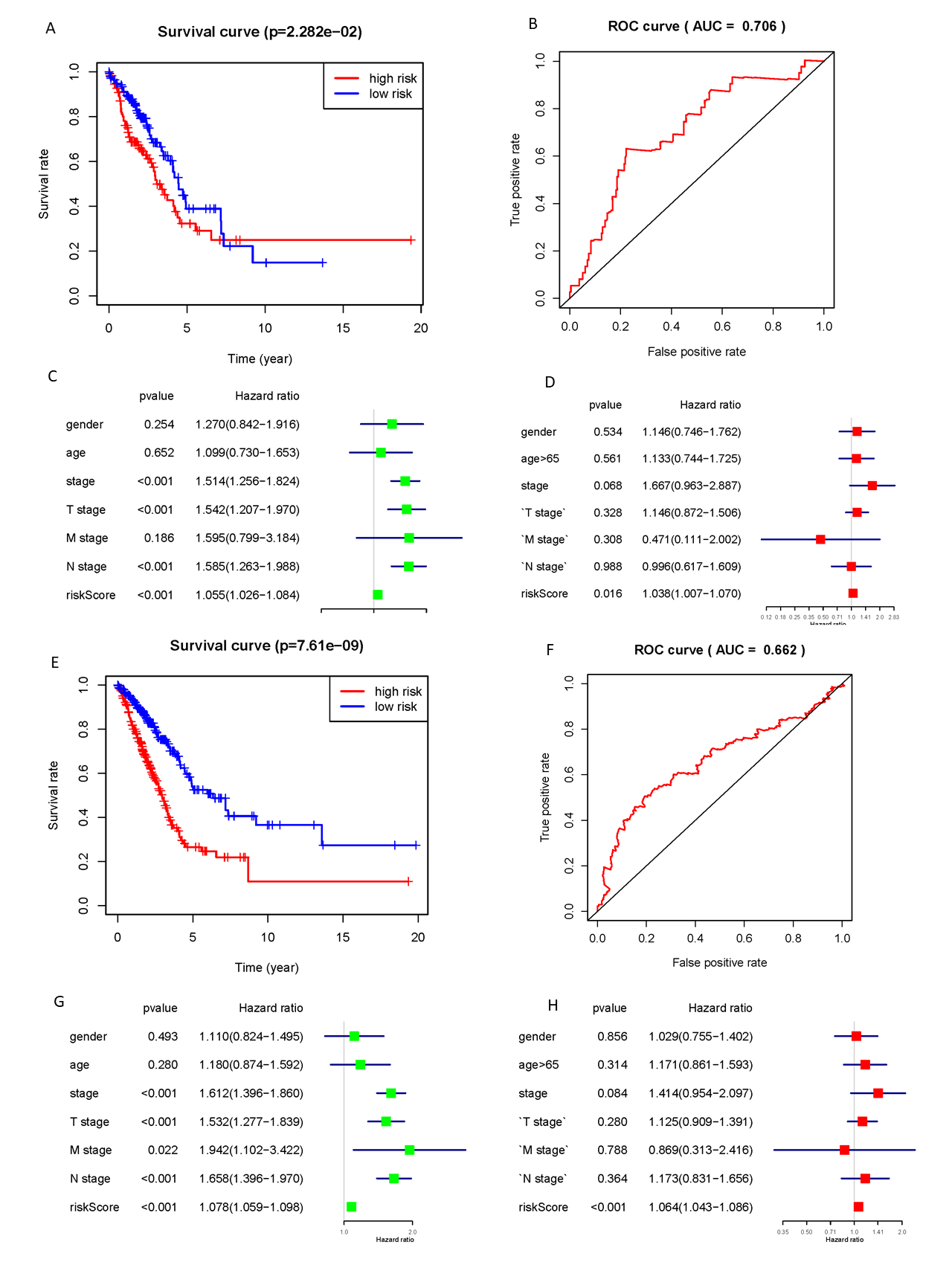


**Figure S1. Validation in the test set and all samples set.** (A-B) The survival and ROC curve in test set. (C-D) Univariate and Multivariate Cox regression analysis of the association between clinicopathological features (including risk score) in test set. (E-F) The survival and ROC curve in all samples set. (G-H) Univariate and Multivariate Cox regression analysis of the association between clinicopathological features (including risk score) in all samples set.


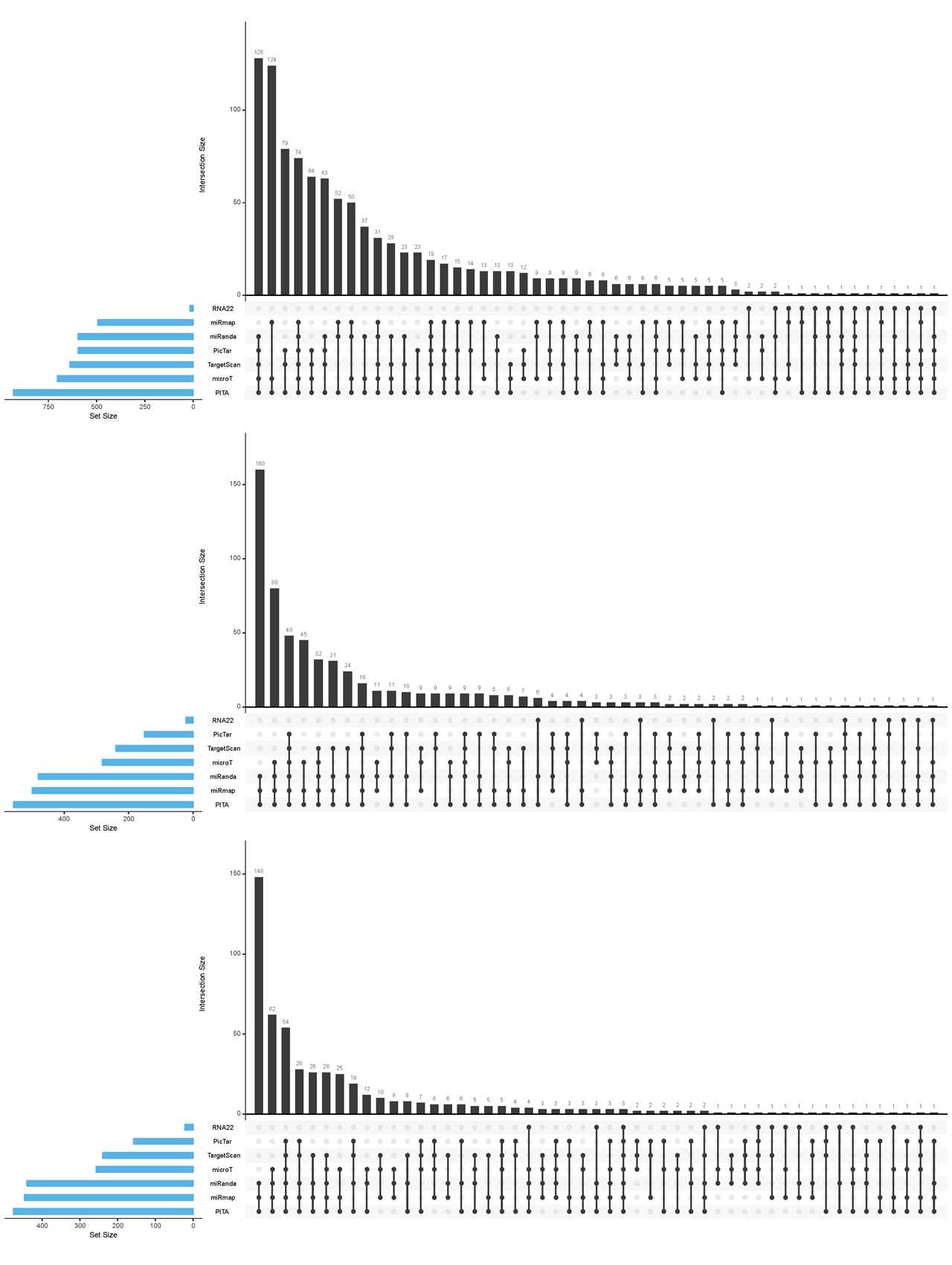


**Figure S2. UpsetView of target genes of hsa-miR-9, hsa-miR-31 and hsa-miR-196b.**
