# Supplementary material for: Hypoxia-associated prognostic markers and competing endogenous RNA coexpression networks in lung adenocarcinoma
Source: Sci Rep. 2022 Dec 9;12:21340. doi: 10.1038/s41598-022-25745-7 (PMC9734750; doi:10.1038/s41598-022-25745-7)
Supplement: Supplementary file 2 — Supplementary Table S1. [file 41598_2022_25745_MOESM2_ESM.docx]

| Gene | HR | 95% CI lower | 95% CI upper | pvalue | Regulated |
| --- | --- | --- | --- | --- | --- |
| CKS2 | 1.3983 | 1.1587 | 1.6873 | 0.0005 | up-regulated |
| RHCG | 1.1368 | 1.0542 | 1.2259 | 0.0009 | up-regulated |
| ORC6L | 1.3534 | 1.1141 | 1.6441 | 0.0023 | up-regulated |
| KIAA0101 | 1.3103 | 1.0943 | 1.5688 | 0.0033 | up-regulated |
| GINS2 | 1.2954 | 1.0861 | 1.5451 | 0.0040 | up-regulated |
| UBE2T | 1.3065 | 1.0806 | 1.5797 | 0.0058 | up-regulated |
| SPC25 | 1.2706 | 1.0688 | 1.5104 | 0.0066 | up-regulated |
| E2F7 | 1.2334 | 1.0570 | 1.4393 | 0.0077 | up-regulated |
| ASF1B | 1.3555 | 1.0806 | 1.7004 | 0.0085 | up-regulated |
| DAPK2 | 0.6193 | 0.5154 | 0.7441 | p<0.0001 | down-regulated |
| IRX5 | 0.7668 | 0.6901 | 0.8519 | p<0.0001 | down-regulated |
| STK33 | 0.7485 | 0.6664 | 0.8408 | p<0.0001 | down-regulated |
| FAM184A | 0.7438 | 0.6561 | 0.8433 | p<0.0001 | down-regulated |
| TMEM125 | 0.7235 | 0.6294 | 0.8317 | p<0.0001 | down-regulated |
| ABAT | 0.6739 | 0.5676 | 0.8001 | p<0.0001 | down-regulated |
| ATP8A1 | 0.6993 | 0.5984 | 0.8173 | p<0.0001 | down-regulated |
| PNMA2 | 0.7572 | 0.6705 | 0.8551 | p<0.0001 | down-regulated |
| HLF | 0.7905 | 0.7131 | 0.8764 | p<0.0001 | down-regulated |
| ST3GAL5 | 0.7134 | 0.6150 | 0.8276 | p<0.0001 | down-regulated |
| ESYT3 | 0.7498 | 0.6605 | 0.8511 | p<0.0001 | down-regulated |
| SLC47A1 | 0.7533 | 0.6644 | 0.8541 | p<0.0001 | down-regulated |
| SFTA3 | 0.8560 | 0.7989 | 0.9171 | p<0.0001 | down-regulated |
| C11orf92 | 0.7861 | 0.7045 | 0.8772 | p<0.0001 | down-regulated |
| CD40LG | 0.7366 | 0.6404 | 0.8473 | p<0.0001 | down-regulated |
| GNMT | 0.7036 | 0.5984 | 0.8274 | p<0.0001 | down-regulated |
| GGTLC1 | 0.8396 | 0.7735 | 0.9113 | p<0.0001 | down-regulated |
| GLS2 | 0.7447 | 0.6476 | 0.8564 | p<0.0001 | down-regulated |
| CLUL1 | 0.7723 | 0.6830 | 0.8732 | p<0.0001 | down-regulated |
| CXCL17 | 0.8662 | 0.8089 | 0.9277 | p<0.0001 | down-regulated |
| DNALI1 | 0.7876 | 0.7028 | 0.8828 | p<0.0001 | down-regulated |
| SPATA18 | 0.7929 | 0.7088 | 0.8869 | p<0.0001 | down-regulated |
| NKX2-1 | 0.8552 | 0.7929 | 0.9224 | 0.0001 | down-regulated |
| SELENBP1 | 0.7630 | 0.6694 | 0.8698 | 0.0001 | down-regulated |
| RIC3 | 0.7741 | 0.6837 | 0.8765 | 0.0001 | down-regulated |
| TDRD10 | 0.7890 | 0.7031 | 0.8853 | 0.0001 | down-regulated |
| IRX2 | 0.8454 | 0.7788 | 0.9177 | 0.0001 | down-regulated |
| NAPSA | 0.8775 | 0.8233 | 0.9354 | 0.0001 | down-regulated |
| SCNN1B | 0.8237 | 0.7489 | 0.9060 | 0.0001 | down-regulated |
| SFTPB | 0.8899 | 0.8403 | 0.9424 | 0.0001 | down-regulated |
| TMPRSS2 | 0.7984 | 0.7144 | 0.8923 | 0.0001 | down-regulated |
| KIAA1324 | 0.8319 | 0.7594 | 0.9114 | 0.0001 | down-regulated |
| FAM189A2 | 0.7920 | 0.7050 | 0.8897 | 0.0001 | down-regulated |
| CD302 | 0.6748 | 0.5540 | 0.8220 | 0.0001 | down-regulated |
| MBL1P | 0.7576 | 0.6588 | 0.8713 | 0.0001 | down-regulated |
| NAPSB | 0.7883 | 0.6993 | 0.8887 | 0.0001 | down-regulated |
| CAPN3 | 0.7469 | 0.6447 | 0.8653 | 0.0001 | down-regulated |
| SFTA1P | 0.8288 | 0.7539 | 0.9112 | 0.0001 | down-regulated |
| C16orf89 | 0.8804 | 0.8253 | 0.9391 | 0.0001 | down-regulated |
| ACSS1 | 0.7441 | 0.6397 | 0.8655 | 0.0001 | down-regulated |
| GPR116 | 0.7796 | 0.6864 | 0.8855 | 0.0001 | down-regulated |
| C11orf16 | 0.8115 | 0.7291 | 0.9032 | 0.0001 | down-regulated |
| ABCA3 | 0.7914 | 0.7017 | 0.8926 | 0.0001 | down-regulated |
| CACNA2D2 | 0.8575 | 0.7917 | 0.9287 | 0.0002 | down-regulated |
| PHYHD1 | 0.8084 | 0.7239 | 0.9028 | 0.0002 | down-regulated |
| VWA2 | 0.7779 | 0.6824 | 0.8866 | 0.0002 | down-regulated |
| CDKL2 | 0.7800 | 0.6850 | 0.8881 | 0.0002 | down-regulated |
| GLB1L3 | 0.8820 | 0.8258 | 0.9420 | 0.0002 | down-regulated |
| CYP4B1 | 0.8801 | 0.8230 | 0.9411 | 0.0002 | down-regulated |
| KIAA2022 | 0.8059 | 0.7196 | 0.9026 | 0.0002 | down-regulated |
| COL4A3 | 0.8416 | 0.7685 | 0.9216 | 0.0002 | down-regulated |
| PRMT8 | 0.8240 | 0.7439 | 0.9128 | 0.0002 | down-regulated |
| C1orf88 | 0.7795 | 0.6833 | 0.8893 | 0.0002 | down-regulated |
| FLJ42875 | 0.8134 | 0.7289 | 0.9077 | 0.0002 | down-regulated |
| PRDM16 | 0.8400 | 0.7653 | 0.9220 | 0.0002 | down-regulated |
| ABCC6 | 0.7749 | 0.6762 | 0.8881 | 0.0002 | down-regulated |
| SCNN1G | 0.8338 | 0.7566 | 0.9189 | 0.0002 | down-regulated |
| VIPR1 | 0.7885 | 0.6944 | 0.8954 | 0.0002 | down-regulated |
| SLC34A2 | 0.8672 | 0.8034 | 0.9361 | 0.0003 | down-regulated |
| ALOX15B | 0.8339 | 0.7554 | 0.9205 | 0.0003 | down-regulated |
| MS4A8B | 0.8757 | 0.8146 | 0.9415 | 0.0003 | down-regulated |
| PCDH20 | 0.8591 | 0.7907 | 0.9334 | 0.0003 | down-regulated |
| ATP13A4 | 0.8591 | 0.7904 | 0.9338 | 0.0004 | down-regulated |
| BTG2 | 0.7214 | 0.6025 | 0.8639 | 0.0004 | down-regulated |
| DNAJB13 | 0.8227 | 0.7386 | 0.9164 | 0.0004 | down-regulated |
| TMEM163 | 0.8247 | 0.7410 | 0.9179 | 0.0004 | down-regulated |
| LMO3 | 0.8700 | 0.8050 | 0.9402 | 0.0004 | down-regulated |
| LOC283174 | 0.8230 | 0.7383 | 0.9174 | 0.0004 | down-regulated |
| SLC5A2 | 0.7692 | 0.6637 | 0.8915 | 0.0005 | down-regulated |
| CX3CR1 | 0.7997 | 0.7052 | 0.9068 | 0.0005 | down-regulated |
| ADHFE1 | 0.7725 | 0.6681 | 0.8933 | 0.0005 | down-regulated |
| CD1E | 0.8428 | 0.7654 | 0.9282 | 0.0005 | down-regulated |
| CYP2B7P1 | 0.8789 | 0.8171 | 0.9454 | 0.0005 | down-regulated |
| PIGR | 0.9022 | 0.8511 | 0.9563 | 0.0005 | down-regulated |
| C6orf138 | 0.8100 | 0.7185 | 0.9132 | 0.0006 | down-regulated |
| GPR98 | 0.8412 | 0.7621 | 0.9285 | 0.0006 | down-regulated |
| CHIA | 0.8741 | 0.8094 | 0.9439 | 0.0006 | down-regulated |
| GAS2L2 | 0.8367 | 0.7554 | 0.9268 | 0.0006 | down-regulated |
| PTGDS | 0.8120 | 0.7204 | 0.9152 | 0.0006 | down-regulated |
| IGFALS | 0.7995 | 0.7029 | 0.9093 | 0.0007 | down-regulated |
| UNC13B | 0.6953 | 0.5640 | 0.8572 | 0.0007 | down-regulated |
| DAAM2 | 0.7990 | 0.7020 | 0.9095 | 0.0007 | down-regulated |
| VSIG2 | 0.8795 | 0.8162 | 0.9478 | 0.0008 | down-regulated |
| PHACTR1 | 0.7571 | 0.6434 | 0.8908 | 0.0008 | down-regulated |
| CLIC6 | 0.8518 | 0.7754 | 0.9357 | 0.0008 | down-regulated |
| MS4A15 | 0.8819 | 0.8191 | 0.9494 | 0.0008 | down-regulated |
| PCP4L1 | 0.8611 | 0.7885 | 0.9403 | 0.0009 | down-regulated |
| CYP4Z1 | 0.7691 | 0.6589 | 0.8978 | 0.0009 | down-regulated |
| CD1C | 0.8437 | 0.7632 | 0.9327 | 0.0009 | down-regulated |
| DLEC1 | 0.8186 | 0.7273 | 0.9212 | 0.0009 | down-regulated |
| FAM149A | 0.7814 | 0.6753 | 0.9041 | 0.0009 | down-regulated |
| AQP3 | 0.8359 | 0.7517 | 0.9295 | 0.0009 | down-regulated |
| SNX30 | 0.7149 | 0.5846 | 0.8742 | 0.0011 | down-regulated |
| C6 | 0.8560 | 0.7798 | 0.9396 | 0.0011 | down-regulated |
| TPPP | 0.8085 | 0.7116 | 0.9186 | 0.0011 | down-regulated |
| CYP4X1 | 0.8418 | 0.7587 | 0.9339 | 0.0012 | down-regulated |
| PLD4 | 0.8278 | 0.7384 | 0.9280 | 0.0012 | down-regulated |
| PLA2G4F | 0.8311 | 0.7424 | 0.9304 | 0.0013 | down-regulated |
| PARM1 | 0.7913 | 0.6857 | 0.9131 | 0.0014 | down-regulated |
| PNPLA7 | 0.8056 | 0.7055 | 0.9199 | 0.0014 | down-regulated |
| DNAH6 | 0.8344 | 0.7464 | 0.9328 | 0.0015 | down-regulated |
| SLC15A2 | 0.8201 | 0.7258 | 0.9267 | 0.0015 | down-regulated |
| ELF5 | 0.8633 | 0.7886 | 0.9451 | 0.0015 | down-regulated |
| NWD1 | 0.8666 | 0.7933 | 0.9466 | 0.0015 | down-regulated |
| TEPP | 0.8299 | 0.7397 | 0.9312 | 0.0015 | down-regulated |
| C2orf40 | 0.8599 | 0.7833 | 0.9439 | 0.0015 | down-regulated |
| B3GALT2 | 0.8121 | 0.7140 | 0.9236 | 0.0015 | down-regulated |
| KLHDC7A | 0.8515 | 0.7706 | 0.9407 | 0.0016 | down-regulated |
| ZNF750 | 0.8619 | 0.7857 | 0.9455 | 0.0016 | down-regulated |
| TMEM132D | 0.8512 | 0.7699 | 0.9412 | 0.0017 | down-regulated |
| HPGDS | 0.8346 | 0.7455 | 0.9343 | 0.0017 | down-regulated |
| ADRB1 | 0.8351 | 0.7461 | 0.9347 | 0.0017 | down-regulated |
| PLIN5 | 0.8010 | 0.6972 | 0.9203 | 0.0017 | down-regulated |
| CD1B | 0.8397 | 0.7526 | 0.9369 | 0.0018 | down-regulated |
| ACSM5 | 0.7993 | 0.6945 | 0.9199 | 0.0018 | down-regulated |
| COL4A4 | 0.8264 | 0.7332 | 0.9314 | 0.0018 | down-regulated |
| SCGB3A1 | 0.9153 | 0.8659 | 0.9676 | 0.0018 | down-regulated |
| EFCAB6 | 0.7691 | 0.6523 | 0.9069 | 0.0018 | down-regulated |
| TMEM130 | 0.8500 | 0.7675 | 0.9413 | 0.0018 | down-regulated |
| ACOXL | 0.8346 | 0.7451 | 0.9350 | 0.0018 | down-regulated |
| SNTN | 0.8749 | 0.8039 | 0.9521 | 0.0020 | down-regulated |
| MYOZ1 | 0.8030 | 0.6988 | 0.9227 | 0.0020 | down-regulated |
| C4orf31 | 0.8630 | 0.7862 | 0.9474 | 0.0020 | down-regulated |
| FCER1A | 0.8687 | 0.7944 | 0.9500 | 0.0021 | down-regulated |
| C9orf135 | 0.8667 | 0.7909 | 0.9496 | 0.0022 | down-regulated |
| SYNE1 | 0.7562 | 0.6326 | 0.9041 | 0.0022 | down-regulated |
| PLA2G12B | 0.8813 | 0.8128 | 0.9556 | 0.0022 | down-regulated |
| BAAT | 0.8547 | 0.7729 | 0.9451 | 0.0022 | down-regulated |
| LOC149620 | 0.8375 | 0.7474 | 0.9384 | 0.0023 | down-regulated |
| HABP2 | 0.8903 | 0.8263 | 0.9593 | 0.0023 | down-regulated |
| KLK11 | 0.9031 | 0.8458 | 0.9643 | 0.0023 | down-regulated |
| SLC26A5 | 0.7911 | 0.6801 | 0.9202 | 0.0024 | down-regulated |
| NR3C2 | 0.8058 | 0.7006 | 0.9267 | 0.0025 | down-regulated |
| CLEC4F | 0.8117 | 0.7091 | 0.9291 | 0.0025 | down-regulated |
| RCAN2 | 0.7819 | 0.6667 | 0.9170 | 0.0025 | down-regulated |
| HLA-DQB2 | 0.8597 | 0.7792 | 0.9485 | 0.0026 | down-regulated |
| CD207 | 0.8831 | 0.8144 | 0.9575 | 0.0026 | down-regulated |
| B3GNT8 | 0.8049 | 0.6988 | 0.9271 | 0.0026 | down-regulated |
| LRRC36 | 0.8199 | 0.7201 | 0.9335 | 0.0027 | down-regulated |
| ITGA9 | 0.8129 | 0.7100 | 0.9308 | 0.0027 | down-regulated |
| CYP4Z2P | 0.7656 | 0.6427 | 0.9120 | 0.0028 | down-regulated |
| PLA2G3 | 0.8629 | 0.7833 | 0.9507 | 0.0028 | down-regulated |
| SULT1C2 | 0.8504 | 0.7645 | 0.9461 | 0.0029 | down-regulated |
| FOLR1 | 0.8828 | 0.8130 | 0.9586 | 0.0030 | down-regulated |
| CRYM | 0.8876 | 0.8203 | 0.9605 | 0.0031 | down-regulated |
| CCDC48 | 0.7925 | 0.6794 | 0.9244 | 0.0031 | down-regulated |
| C9orf152 | 0.8543 | 0.7693 | 0.9487 | 0.0032 | down-regulated |
| IGSF9B | 0.8459 | 0.7560 | 0.9465 | 0.0035 | down-regulated |
| KLF15 | 0.8125 | 0.7067 | 0.9341 | 0.0035 | down-regulated |
| RNASE1 | 0.8289 | 0.7305 | 0.9405 | 0.0036 | down-regulated |
| SCUBE2 | 0.8458 | 0.7556 | 0.9468 | 0.0036 | down-regulated |
| SFTPD | 0.9005 | 0.8391 | 0.9664 | 0.0036 | down-regulated |
| B3GAT1 | 0.8597 | 0.7762 | 0.9522 | 0.0037 | down-regulated |
| PRICKLE4 | 0.8037 | 0.6934 | 0.9317 | 0.0037 | down-regulated |
| CTSH | 0.7917 | 0.6749 | 0.9286 | 0.0041 | down-regulated |
| DNASE1L3 | 0.8523 | 0.7641 | 0.9507 | 0.0041 | down-regulated |
| SUSD2 | 0.8742 | 0.7973 | 0.9584 | 0.0042 | down-regulated |
| C4BPA | 0.9011 | 0.8391 | 0.9676 | 0.0042 | down-regulated |
| CCDC108 | 0.8664 | 0.7851 | 0.9561 | 0.0043 | down-regulated |
| PGM5 | 0.8202 | 0.7158 | 0.9399 | 0.0043 | down-regulated |
| VWA3A | 0.8642 | 0.7815 | 0.9556 | 0.0044 | down-regulated |
| GPR133 | 0.8608 | 0.7763 | 0.9545 | 0.0045 | down-regulated |
| SLC1A7 | 0.8878 | 0.8179 | 0.9638 | 0.0045 | down-regulated |
| CGNL1 | 0.7457 | 0.6082 | 0.9143 | 0.0048 | down-regulated |
| MYBPHL | 0.8788 | 0.8031 | 0.9616 | 0.0049 | down-regulated |
| HSD17B13 | 0.8393 | 0.7425 | 0.9486 | 0.0051 | down-regulated |
| BTNL9 | 0.8206 | 0.7143 | 0.9428 | 0.0052 | down-regulated |
| CD1A | 0.8879 | 0.8167 | 0.9653 | 0.0053 | down-regulated |
| KCNJ11 | 0.8218 | 0.7157 | 0.9436 | 0.0054 | down-regulated |
| C1orf168 | 0.8734 | 0.7939 | 0.9609 | 0.0055 | down-regulated |
| C20orf56 | 0.8998 | 0.8353 | 0.9694 | 0.0055 | down-regulated |
| GFRA1 | 0.8701 | 0.7887 | 0.9599 | 0.0055 | down-regulated |
| C5orf49 | 0.8567 | 0.7680 | 0.9556 | 0.0055 | down-regulated |
| FCGBP | 0.8726 | 0.7922 | 0.9612 | 0.0057 | down-regulated |
| CFTR | 0.8968 | 0.8300 | 0.9690 | 0.0058 | down-regulated |
| C14orf64 | 0.8413 | 0.7439 | 0.9514 | 0.0059 | down-regulated |
| C5orf38 | 0.8809 | 0.8049 | 0.9642 | 0.0059 | down-regulated |
| LOC145837 | 0.8962 | 0.8285 | 0.9694 | 0.0062 | down-regulated |
| FXYD1 | 0.8594 | 0.7705 | 0.9585 | 0.0065 | down-regulated |
| BMP3 | 0.8916 | 0.8207 | 0.9687 | 0.0067 | down-regulated |
| SLC16A11 | 0.8274 | 0.7214 | 0.9489 | 0.0067 | down-regulated |
| NR0B2 | 0.8892 | 0.8165 | 0.9683 | 0.0069 | down-regulated |
| P2RY12 | 0.8456 | 0.7483 | 0.9555 | 0.0071 | down-regulated |
| HSD17B6 | 0.8642 | 0.7771 | 0.9611 | 0.0071 | down-regulated |
| HOPX | 0.8763 | 0.7957 | 0.9650 | 0.0073 | down-regulated |
| CACNA1D | 0.8439 | 0.7454 | 0.9556 | 0.0074 | down-regulated |
| CLEC3B | 0.8474 | 0.7505 | 0.9567 | 0.0075 | down-regulated |
| PRR15L | 0.8362 | 0.7326 | 0.9545 | 0.0081 | down-regulated |
| OGN | 0.8817 | 0.8027 | 0.9683 | 0.0085 | down-regulated |
| MST1P2 | 0.8547 | 0.7603 | 0.9608 | 0.0085 | down-regulated |
| CA3 | 0.8443 | 0.7442 | 0.9579 | 0.0086 | down-regulated |
| AMY2B | 0.8288 | 0.7201 | 0.9538 | 0.0088 | down-regulated |
| ADAMTS8 | 0.8695 | 0.7829 | 0.9657 | 0.0090 | down-regulated |
| C1orf186 | 0.8328 | 0.7258 | 0.9554 | 0.0091 | down-regulated |
| SLC14A1 | 0.8172 | 0.7020 | 0.9513 | 0.0092 | down-regulated |
| NFIX | 0.7939 | 0.6669 | 0.9449 | 0.0094 | down-regulated |
| CA10 | 0.8997 | 0.8304 | 0.9747 | 0.0097 | down-regulated |
| EDA2R | 0.8592 | 0.7659 | 0.9640 | 0.0097 | down-regulated |
| SEC14L3 | 0.8382 | 0.7332 | 0.9583 | 0.0098 | down-regulated |
| ABI3BP | 0.8528 | 0.7556 | 0.9625 | 0.0099 | down-regulated |
| CPAMD8 | 0.8690 | 0.7811 | 0.9669 | 0.0099 | down-regulated |

**Table S1. Prognosis-related DEGs in breast cancer.**
