# Supplementary material for: Hypoxia-associated prognostic markers and competing endogenous RNA coexpression networks in lung adenocarcinoma
Source: Sci Rep. 2022 Dec 9;12:21340. doi: 10.1038/s41598-022-25745-7 (PMC9734750; doi:10.1038/s41598-022-25745-7)
Supplement: Supplementary file 3 — Supplementary Table S2. [file 41598_2022_25745_MOESM3_ESM.docx]

| MicroRNAs | logFC | P Value | regulated |
| --- | --- | --- | --- |
| hsa-miR-105-5p | 2.194471419 | 8.34E-09 | Up-Regulated |
| hsa-miR-767-5p | 1.776191914 | 1.16E-05 | Up-Regulated |
| hsa-miR-196b-5p | 1.632260928 | 7.40E-12 | Up-Regulated |
| hsa-miR-210-3p | 1.545310976 | 1.42E-18 | Up-Regulated |
| hsa-miR-9-5p | 1.425485581 | 4.33E-09 | Up-Regulated |
| hsa-miR-31-5p | 1.308636842 | 1.64E-09 | Up-Regulated |
| hsa-miR-675-3p | 1.239961044 | 6.27E-08 | Up-Regulated |
| hsa-miR-31-3p | 1.160429126 | 7.70E-10 | Up-Regulated |
| hsa-miR-1269a | 1.088752269 | 0.007908634 | Up-Regulated |
| hsa-miR-205-5p | 1.071114903 | 0.000960554 | Up-Regulated |
| hsa-miR-6087 | 1.064705029 | 7.15E-17 | Up-Regulated |
| hsa-miR-3065-3p | -1.192209349 | 2.79E-13 | Down-Regulated |

**Table S2. Hypoxia associated dysregulated microRNAs in LUAD.**
